# Exploratory study of the long-term footprint of deep brain stimulation on brain metabolism and neuroplasticity in an animal model of obesity

**DOI:** 10.1038/s41598-021-82987-7

**Published:** 2021-03-10

**Authors:** Marta Casquero-Veiga, Clara Bueno-Fernandez, Diego Romero-Miguel, Nicolás Lamanna-Rama, Juan Nacher, Manuel Desco, María Luisa Soto-Montenegro

**Affiliations:** 1 Laboratorio de Imagen Médica, Instituto de Investigación Sanitaria Gregorio Marañón, Madrid, Spain; 2 Centro de Investigación Biomédica en Red de Salud Mental, Madrid, Spain; 3 Neurobiology Unit, Cell Biology Department, Interdisciplinary Research Structure for Biotechnology and Biomedicine (BIOTECMED), Universitat de València, Madrid, Spain; 4 Fundación Investigación Hospital Clínico de Valencia, INCLIVA, Madrid, Spain; 5 Departamento de Bioingeniería e Ingeniería Aeroespacial, Universidad Carlos III de Madrid, Madrid, Spain; 6 Centro Nacional de Investigaciones Cardiovasculares, Madrid, Spain

**Keywords:** Neuroscience, Molecular neuroscience, Synaptic plasticity, Preclinical research, Translational research

## Abstract

Deep brain stimulation (DBS) is a powerful neurostimulation therapy proposed for the treatment of several neuropsychiatric disorders. However, DBS mechanism of action remains unclear, being its effects on brain dynamics of particular interest. Specifically, DBS reversibility is a major point of debate. Preclinical studies in obesity showed that the stimulation of the lateral hypothalamus (LH) and nucleus accumbens (NAcc), brain centers involved in satiety and reward circuits, are able to modulate the activity of brain structures impaired in this pathology. Nevertheless, the long-term persistence of this modulation after DBS withdrawal was unexplored. Here we examine the in vivo presence of such changes 1 month after LH- and NAcc-DBS, along with differences in synaptic plasticity, following an exploratory approach. Thus, both stimulated and non-stimulated animals with electrodes in the NAcc showed a common pattern of brain metabolism modulation, presumably derived from the electrodes’ presence. In contrast, animals stimulated in the LH showed a relative metabolic invariance, and a reduction of neuroplasticity molecules, evidencing long-lasting neural changes. Our findings suggest that the reversibility or persistence of DBS modulation in the long-term depends on the selected DBS target. Therefore, the DBS footprint would be influenced by the stability achieved in the neural network involved during the stimulation.

## Introduction

Deep brain stimulation (DBS) has elicited major interest in the fields of neurology and psychiatry since the mid-twentieth century as a promising alternative to the ablative neurosurgical procedures^1^. From the early 50s to the late 70s, pioneer researchers such as Delgado, Bekthereva, Sem-Jacobsen and Cooper, first recognized the therapeutic potential of DBS^1–3^. However, it was not until 1987 that Benabid and Pollack discoveries^4^ established DBS as an effective treatment for movement disorders. This revolutionary success led to an increase of advocates for the implementation of DBS to treat psychiatric conditions, which contrasted with a general skepticism based on the controversial past of lesioning psychosurgery^1^. Despite its polemic beginnings, DBS benefits^5–7^ continue inspiring extensive research work in the psychiatric field^8^. In this sense, besides its well-recognized role in treating motor diseases^9^, DBS has already received FDA approval for obsessive–compulsive disorder (OCD) and focal epilepsy treatment^8^.

Regardless of its undoubted efficacy, there are a wide number of unanswered questions concerning DBS mechanism of action and long-term impact. Several theories have arisen in the last decades, aiming to unify the brain effects induced by DBS (see^10^ for a review). This lack of consensus responds to the dependency of the observed effects on the brain target and disease under study. Furthermore, there are few studies describing the permanence of physiological effects after completing a DBS protocol^11–14^. In this respect, the traditional conception attributes to DBS a reversible nature, describing a rebound of the symptoms after the stimulation withdrawal^15,16^. However, some studies showed a lack of reinstatement of pathological symptoms following DBS removal (even after a long-term follow-up^11,17^), or a paradoxical improvement of the patient’s symptomatology^13^. Consequently, there is still no obvious evidence of the underlying phenomena, while the study of the neural consequences during and after DBS treatment seems crucial to spread its application to other pathologies.

In this sense, treatment-resistant obesity appears as a candidate disease to benefit from this therapy^18^. Obesity etiology involves a substantial neuropsychiatric component in which homeostatic and reward brain centers play crucial roles. Food intake activates the brain circuits related to reward, whose alteration leads to the adoption of compulsive behaviors, in much the same way as in drug addiction^19^. Therefore, obesity can somewhat be considered a ‘food addiction’^20,21^.

Among the considered DBS targets against obesity, the hypothalamus emerges as a good candidate since it harbors the appetite (lateral hypothalamus, LH) and satiety brain cores (ventromedial hypothalamus, VMH)^22^. In fact, a pilot study^23^ showed the safety of applying LH-DBS for treatment-resistant obesity and reported a significant reduction in body weight in two out of three patients studied. Further research showed that LH-DBS increased the resting metabolic rate of two patients with treatment refractory obesity^24^, proposing a potential mechanism underlying the DBS efficacy. Furthermore, given that enhanced anticipation of the food rewarding properties may partially drive to its consumption^25^, reward-related areas could also become successful targets for DBS. A major brain core belonging to this system is the nucleus accumbens (NAcc), whose stimulation could counteract the reward feeling associated with food intake and theoretically lead to weight reduction^22^. Accordingly, four clinical trials testing the efficacy of high frequency NAcc-DBS reported significant weight reductions in those patients who finished the follow-up period^26–29^.

In previous studies, we showed that LH-DBS in a rat model of genetic obesity (Zucker rat) induced weight gain reductions and metabolic changes in brain regions related to the control of food intake^30^. On the contrary, NAcc-DBS in the same model did not affect the weight gain, but led to metabolic changes in cognitive- and reward-related brain regions^31^. In addition, despite the proven effects of DBS on neuronal activity^32,33^, very few studies have explored its effects on synaptic structural plasticity, and they mainly focused on the quantification of synapses via synaptophysin^34–36^. Here, we have a two-fold aim: (1) to examine which brain changes remained 1 month after the end of LH- and NAcc-DBS by means of positron emission tomography (PET) with [^18^F]-fluorodeoxyglucose studies, and (2) to evaluate the density of excitatory and inhibitory synaptic markers (VGAT and VGLUT1), together with an assessment of a plasticity related molecule PSA-NCAM (polysialylated form of the neural cell adhesion molecule), in two regions modulated by LH- and NAcc-DBS (i.e. the hippocampus and entorhinal cortex) at this time point.

## Methods

Our study resulted from a 1-month follow-up performed to the same animals included in previous works by our group, in which we addressed the effects of a 15-days intermittent protocol of high frequency DBS applied in LH^30^ or NAcc^31^ in a genetic animal model of obesity.

### Animals

**Figure 1:**
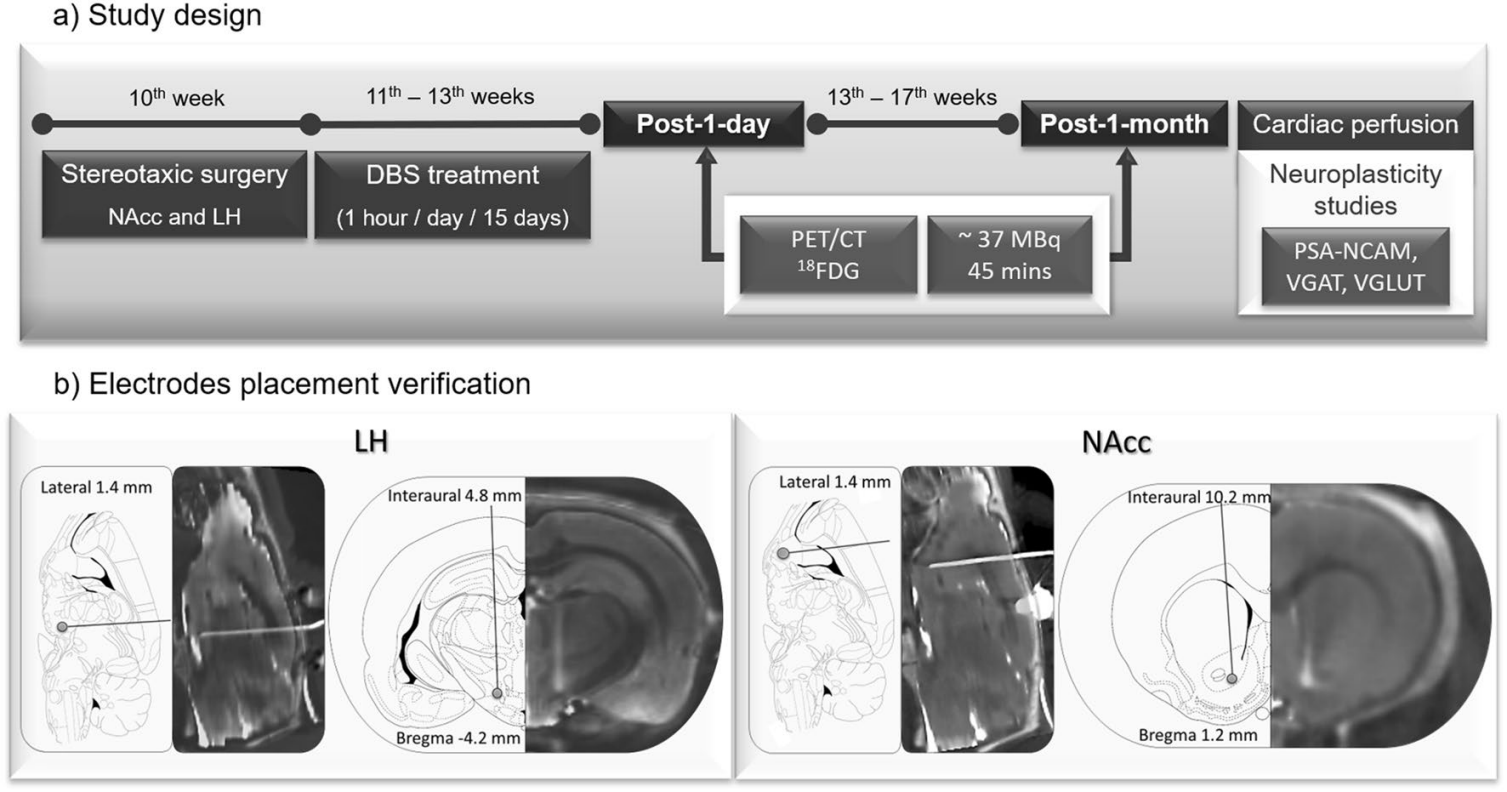
(**a**) Study design. Design of the experimental procedures performed during the study. (**b**) Electrodes placement verification. Sagittal and axial views of a CT scan of an animal registered to the MR template next to the correspondent slice from^38^ to verify the correct electrode location in LH (left) and NAcc (right).

Adult male Zucker rats (fa/fa-, Charles Rivers Laboratories, Spain) (10-week old) (N = 25) were housed individually in a temperature- and humidity-controlled room on a 12 h dark/light cycle with food (standard laboratory chow) and water available ad libitum. Prior to the PET studies, animals were deprived of food but allowed free access to water for 6–8 h. Figure 1a summarizes the complete study design.

All experimental animal procedures were conducted according to European Communities Council Directive 2010/63/EU and approved by the Ethics Committee for Animal Experimentation of Hospital Gregorio Marañón and Madrid Regional Government (PROEX 026/16).

### Surgeries

Animals underwent stereotactic surgery when they reached 10 weeks of age under a mixture of ketamine/xylazine (100/10 mg/kg). Concentric bipolar platinum-iridium electrodes (Bilaney Consultants GmbH, Germany) were bilaterally implanted to target the NAcc (+ 1.2 mm posterior and + 1.5 mm lateral from bregma, − 8.2 mm ventral from the dura)^37^ (N = 15) and the LH (− 4.0 mm posterior and + 1.6 mm lateral from bregma, − 8.2 mm ventral from the dura)^37^ (N = 10). Acrylic dental cement (Technovit®, Heraeus-Kulzer, Germany) was applied to fix the electrodes to the skull. A 3-days postoperative protocol was followed by providing ceftriaxone (100 mg/kg IM) and buprenorphine (0.1 mg/kg IP) doses to prevent infections and pain. The correct electrode location was verified by the acquisition of a computerized tomography (CT) image (Fig. 1b).

### DBS protocol

The stimulation protocol started 7 days after surgery to ensure enough time for recovering. 4 groups of animals were established: NAcc-sham (N = 9) and LH-sham (N = 4) (surgery but no stimulation), and NAcc-DBS (N = 6) and LH-DBS (N = 6) (surgery plus stimulation). An isolated stimulator device (CS 120 8i, CIBERTEC S.A., Spain) delivered the stimulation at a constant current of 150 µA (130 Hz) and a pulse width of 100 µs. Stimulation was applied for 1 h/day over 15 days (see^30,31^ for further details).

### Imaging studies

Acquisition of static PET studies was performed 1 day and 1 month after the end of the DBS protocol, using a small-animal PET/CT scanner (ARGUS PET/CT, SEDECAL, Spain). 2-deoxy-2-[^18^F]fluoro-D-glucose (FDG) (~ 37 MBq) was intravenously injected through the tail vein, allowing a total uptake period of 45 min. Each PET acquisition lasted 45 min. CT images were also obtained with the same scanner, and they were used to ensure the absence of electrode displacement along the study (Supplementary Figs. 1 and 2). Furthermore, an anatomical template for the voxelwise PET study was obtained by acquiring a T2 spin-echo magnetic resonance image (MRI) from a naïve animal with a 7-T Biospec 70/20 scanner (Bruker, Germany). Acquisition and reconstruction protocols have been thoroughly described elsewhere^30,31^.

### Immunofluorescence studies

At the end of post-1-month PET studies, all animals were perfused with a 4% paraformaldehyde solution, and their brains were removed and stored under cryoprotection (30% sucrose in PBS 0.1, 48 h) until used. Brain tissue was cut in 50 µm-thick coronal sections using a freezing-sliding microtome (LEICA SM2010R, Leica, Germany) and collected in 10 subseries. Free-floating sections were used for immunohistochemistry as previously described^38^, using the following primary antibodies: polyclonal guinea pig anti-vesicular glutamate transporter 1 (VGLUT1, 1:2000, Merck-Millipore, Spain), monoclonal rabbit anti-vesicular GABA transporter (VGAT, 1:1000, Synaptic Systems, Germany) and monoclonal IgM mouse anti-PSA-NCAM (PSA-NCAM, 1:700, Merck-Millipore, Spain).

### Data processing and statistical analysis

#### PET data

PET data followed a preprocessing registration protocol previously described^31^. We performed an iterative voxel value normalization method^39,40^. This protocol provided a “non-significant area” (NSA), which served as a normalization region to standardize the intensity values in our PET studies (see Supplementary Fig. 3). Then, an analysis throughout the entire brain was performed to evaluate regional changes in brain glucose metabolism. Images were studied by voxel-wise analyses using SPM12 software (http://www.fil.ion.ucl.ac.uk/spm/software/spm12/) with a multifactorial ANOVA (p < 0.01, uncorrected), considering group (sham, DBS) and time point (post-1-day, post-1-month) as study factors. In order to reduce type I error, only significant regions larger than 50 activated adjacent voxels were considered. A cluster-based multiple-comparison correction (p < 0.05) was applied to limit type II errors^41^, but no further corrections for multiple comparisons were applied related due to the exploratory nature of the study.

### Immunofluorescence data

The images used for the analysis of neuropil puncta expressing VGAT, VGLUT1 and PSA-NCAM were obtained with a confocal microscope (Leica TCS SPE). We analyzed different layers of the hippocampal CA1 field (pyramidale, radiatum; − 3.6 mm posterior and 1.5–4 mm lateral from Bregma) and the lateral entorhinal cortex (layer I-II, layer III, deep layer (V and VI); − 5.2 mm posterior and 6.7–8 mm lateral from Bregma). These regions were selected due to the effect of NAcc-DBS on hippocampal activity and neurogenesis promotion^42^, the effect of LH-DBS on hippocampal brain metabolism^30^, and its proposed benefits on memory dependent on these brain regions^43^.

Confocal z-stacks covering the whole depth of the sections were taken with 1 μm step size and only subsets of confocal planes with the optimal penetration level for each antibody were selected. On these planes, small regions of the neuropil (890 μm^2^) were selected for analysis, in order to avoid blood vessels and cell somata. Images were processed using FIJI/ImageJ software^44^ as previously described^38^. The number of the resulting dots per region was counted. Statistics were performed using the number of animals as the “n”. The data were subjected to Student’s T-test analysis and figures were plotted with GraphPad Prism 8.

### Ethical approval

All applicable international, national, and/or institutional guidelines for the care and use of animals were followed.

## Results

### ‘In vivo’ study of the DBS metabolic footprint

#### LH-DBS produces long-term regional changes after stimulation withdrawal

**Figure 2:**
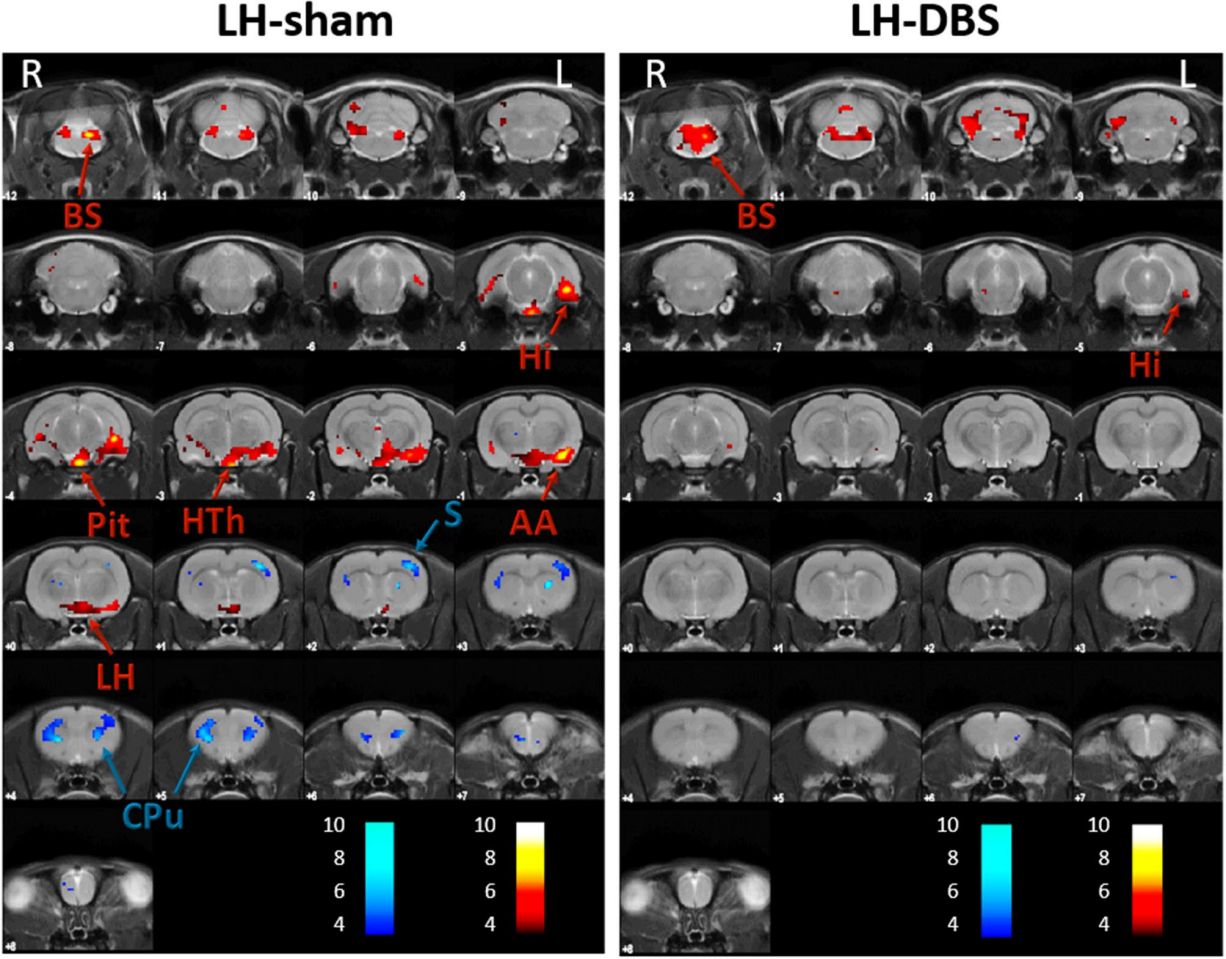
Persistence of DBS effects on brain metabolism 1 month after finishing LH-DBS treatment. Colored PET overlays on the MR reference represent the T-maps resulting from the post-1-month vs. post-1-day comparisons, indicating increased (hot colors) or decreased FDG uptake (cold colors), in LH-sham (left) and LH-DBS (right) animals, respectively. The color bars represent the T values scale corresponding to the PET overlays intensity. Hemispheres: *Left* (L), *Right* (R). *AA* amygdala, *BS* brainstem, *Cb* cerebellum, *CPu* caudate-putamen, *Hi* hippocampus, *HTh* hypothalamus, *LH* lateral hypothalamus, *Pit* pituitary gland S: somatosensory cortex.

LH-sham and LH-DBS animals showed important differences in brain metabolism which persisted 1 month after DBS withdrawal (Table 1a,b) (Fig. 2). Thus, LH-sham group showed a bilateral reduction in brain metabolism in the caudate-putamen and somatosensory cortex, while increased FDG uptake was evident in different portions of the LH, amygdala, pituitary gland, ventral hippocampus and brainstem at post-1-month compared with post-1-day. On the contrary, LH-DBS animals showed very few changes, with increased FDG uptake in the hippocampus, brainstem and cerebellum at post-1-month compared with post-1-day.

**Table 1:** Permanence of brain metabolic changes 1 month after stimulation.

ROI	Side	T	k	↓/↑	p_UNC_ peak level	p_UNC_ cluster level	p_FWE_ cluster level
**(A) LH-sham**							
CPu-S	L	10.05	888	↓	< 0.001	< 0.001	< 0.001
Hi	L	9.04	2145	↑	< 0.001	< 0.001	< 0.001
Bs	L-R	4.09			< 0.001		
AA	L	3.69			< 0.001		
**(B) LH-DBS**							
BS-Cb	L-R	3.98	1055	↑	< 0.001	< 0.001	< 0.001
		2.59			< 0.001		
Hi	L	5.30	93	↑	< 0.001	0.038	0.350
**(C) NAcc-sham**							
SC-IC	R	8.14	237	↓	< 0.001	0.010	0.020
OC	R	6.29	378	↓	< 0,001	0.002	0.011
cc	L	5.37	240	↓	< 0.001	0.009	0.020
BS	L-R	5.02	216	↑	< 0.001	0.013	0.099
HTh	L-R	4.68	291	↑	< 0.001	0.005	0.039
**(D) NAcc-DBS**							
SC-IC	R	6.90	133	↓	< 0.001	0.043	0.292
Ect	L	6.86	1814	↓	< 0.001	< 0.001	< 0.001
S-cc-NAcc	L-R	5.92			< 0.001		
HTh	L-R	5.86	582	↑	< 0.001	< 0.001	0.002
BS	L-R	5.61	320	↑	< 0.001	0.004	0.028

*AA* amygdala, *BS* brainstem, *Cb* cerebellum, *cc* corpus callosum, *CPu* caudate-putamen, *Ect* ectorhinal cortex, *Hi* hippocampus, *HTh* hypothalamus, *IC* inferior colliculus, *LH* lateral hypothalamus, *NAcc* nucleus accumbens, *OC* orbital cortex, *Pit* pituitary gland *S* somatosensory cortex, *SC* superior colliculus, *ROI* region of interest. Side: Right (R) and Left (L). *T* t value, *k* cluster size. Glucose metabolism: Increase (↑) and Decrease (↓). *p*_*UNC*_ p value uncorrected, *FWE* family wise error correction.

#### NAcc-DBS effects barely persist over time

**Figure 3:**
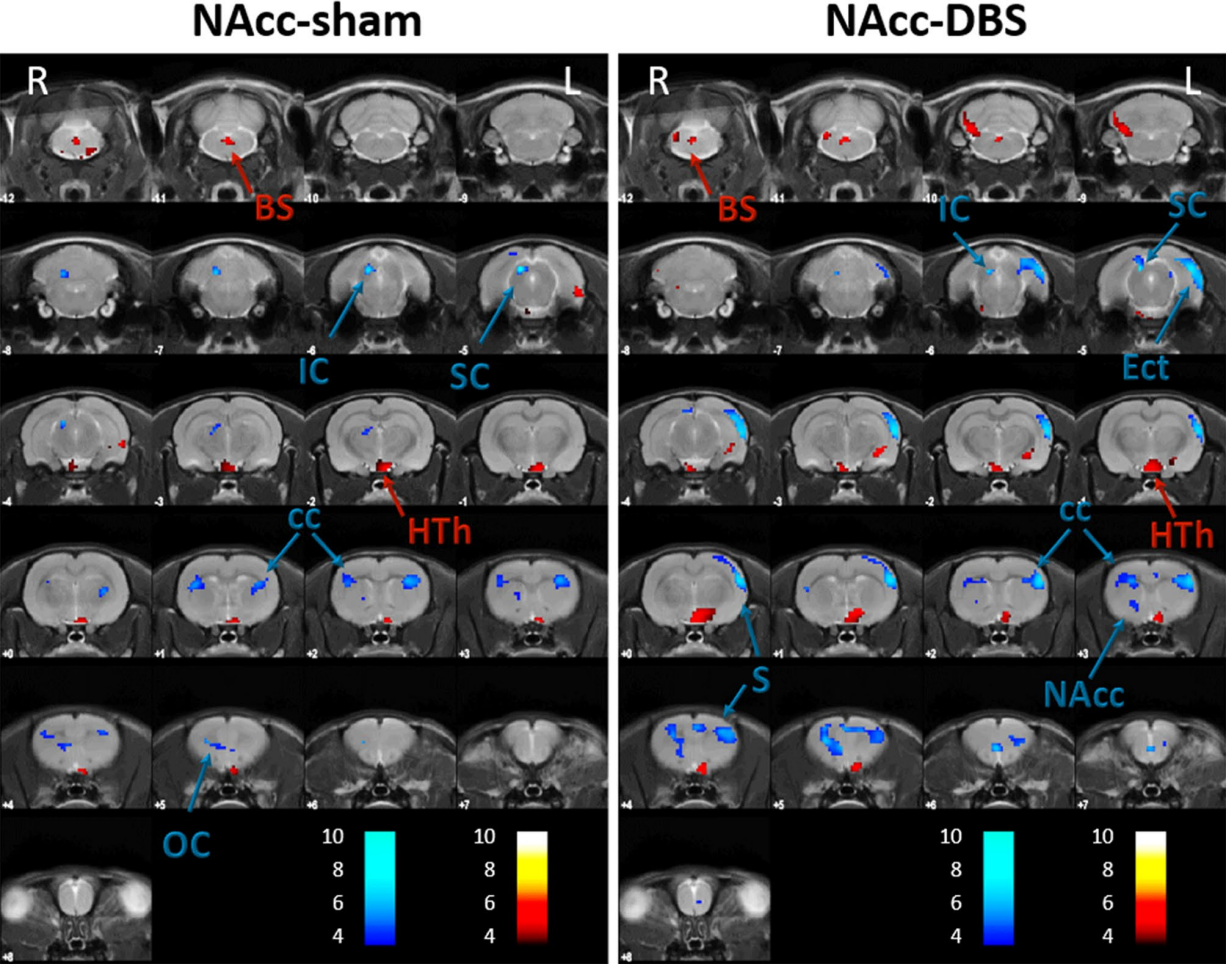
Persistence of DBS effects on brain metabolism 1 month after finishing NAcc-DBS treatment. Colored PET overlays on the MR reference represent the T-maps resulting from the post-1-month vs. post-1-day comparisons, indicating increased (hot colors) or decreased FDG uptake (cold colors), in NAcc-sham (left) and NAcc-DBS (right) animals, respectively. The color bars represent the T values scale corresponding to the PET overlays intensity. Hemispheres: *Left* (L), *Right* (R). *BS* brainstem, *cc* corpus callosum, *Ect* ectorhinal cortex, *HTh* hypothalamus, *IC* inferior colliculus, *NAcc* nucleus accumbens, *OC* orbital cortex, *S* somatosensory cortex, *SC* superior colliculus.

In contrast with LH-groups, NAcc-sham and NAcc-DBS animals did not differ as much when comparing brain metabolic changes 1 day and 1 month after the DBS protocol ending (Table 1c,d) (Fig. 3).

In NAcc-sham animals, a reduction in FDG uptake was detected in the orbital cortex, corpus callosum, inferior and superior colliculi, as well as increases in hypothalamus and brainstem 1 month after the first PET study. Similarly, NAcc-DBS animals showed a glucose metabolism decrease in somatosensory cortex, NAcc, corpus callosum, ectorhinal cortex, inferior and superior colliculi, together with an increase in hypothalamus and brainstem at post-1-month compared with post-1-day.

### Density of PSA-NCAM, VGLUT1 and VGAT expressing puncta in the neuropil of the hippocampus and the entorhinal cortex

#### DBS alters the density of PSA-NCAM expressing puncta

**Figure 4:**
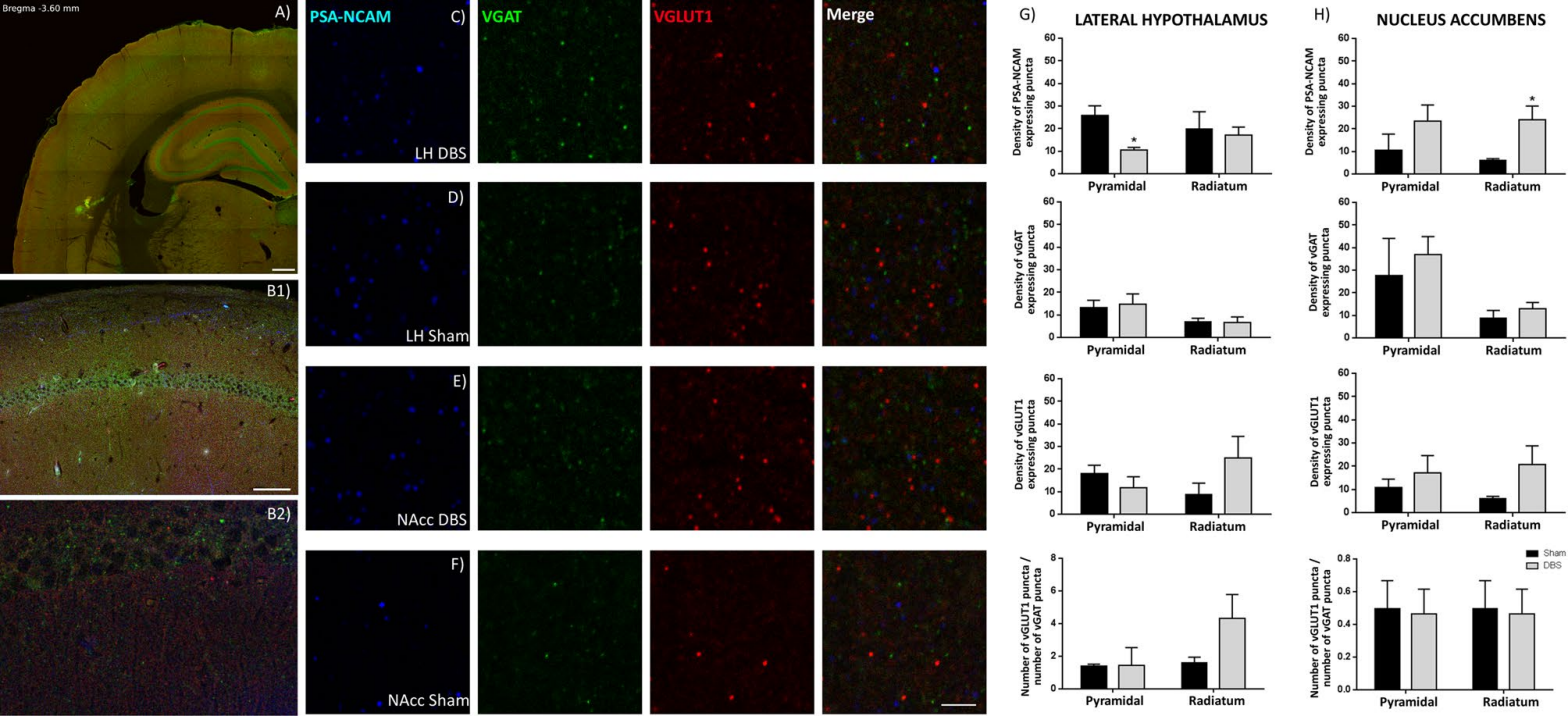
DBS effects on synaptic plasticity in the hippocampus. (**A**) Panoramic view of the dorsal hippocampus immunostained for VGAT and VGLUT1. (**B1**) Panoramic view of CA1 region. (**B2**) Detailed view of the *strata pyramidale* and *radiatum*. (**C**,**D**) Expression of molecules related to neuronal plasticity in the *stratum pyramidal*e of LH stimulated animals (**C**), and LH-sham animals (**D**). (**E**,**F**) Expression of molecules related to neuronal plasticity in the *stratum pyramidal*e of NAcc stimulated animals (**E**) and in NAcc-sham animals (**F**). (**G**,**H**) Graphs showing the density of puncta (number of puncta/900 µm^2^) expressing PSA-NCAM, VGAT, VGLUT1 and the E/I balance in the rats receiving stimulation in the LH (**G**) and the NAcc (**H**). Student’s T-test, *p*-values: * < 0.05). *VGAT* vesicular GABA transporter; *VGLUT* vesicular glutamate transporter 1. Scale bar 500 µm for (**A**), 50 µm for (**B1**), 20 µm for (**B2**), 3 µm for (**C**–**F**).

**Figure 5:**
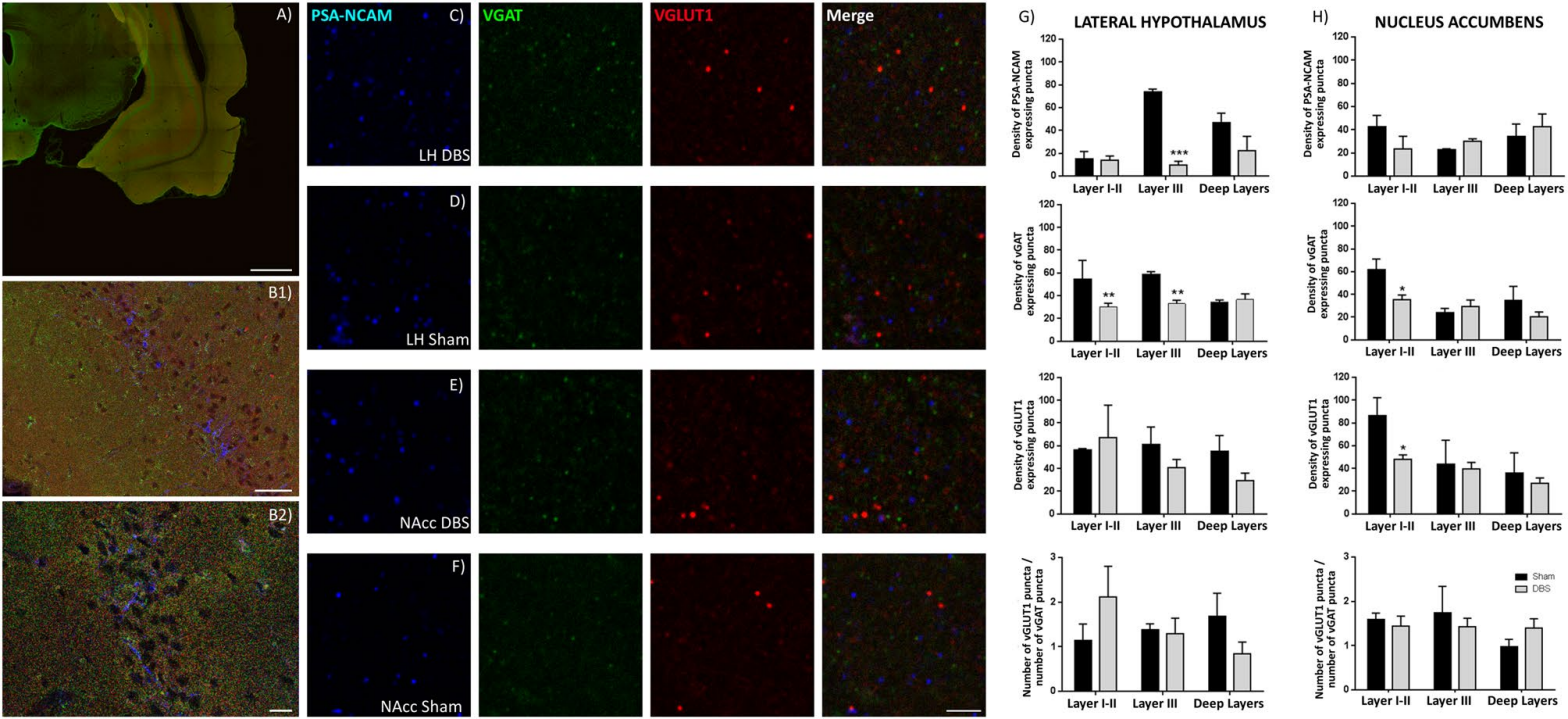
DBS effects on synaptic plasticity in the entorhinal cortex. (**A**) Panoramic view of the entorhinal cortex and ventral hippocampus immunostained for VGAT and VGLUT1. (**B1**) Panoramic view of the lateral entorhinal cortex. (**B2**) Detailed view of the layer III of the lateral entorhinal cortex. (**C**,**D**) Expression of molecules related to neuronal plasticity in layer III of LH stimulated animals (**C**) and in LH-sham animals (**D**). (**E**,**F**) Expression of molecules related to neuronal plasticity in layer III in NAcc-stimulated animals (**E**) and NAcc-sham animals (**F**). (**G**,**H**) Graphs showing the density of puncta (number of puncta/900 µm^2^) expressing PSA-NCAM, VGAT, VGLUT1 and the E/I balance in the rats receiving stimulation in the LH (**G**) and the NAcc (**H**). Student’s T-test, p-values: * < 0.05, ** < 0.01, *** < 0.001. *VGAT* vesicular GABA transporter; *VGLUT* vesicular glutamate transporter 1. Scale bar 500 µm for (**A**), 50 µm for (**B1**), 20 µm for (**B2**), 3 µm for (**C**–**F**).

Animals stimulated in LH showed a significant decrease in the density of puncta-expressing PSA-NCAM in the *stratum pyramidale* (p < 0.05) and a non-significant trend towards a decrease in the *stratum radiatum* compared to sham animals (Fig. 4). By contrast, we found a significant increase in the density of PSA-NCAM + puncta in the *stratum radiatum* (p < 0.05) and a non-significant trend towards an increase in the *stratum pyramidale* in NAcc-DBS animals (Fig. 4). The density of puncta expressing PSA-NCAM in the entorhinal cortex was significantly decreased in the layer III (p < 0.001) of LH-DBS animals when compared with sham animals (Fig. 5), while no significant changes were observed between NAcc groups.

#### DBS alters the density of VGAT and VGLUT1 expressing puncta in the entorhinal cortex, but not in the hippocampus

LH-DBS animals showed a significant decrease in the density of puncta expressing VGAT (p < 0.01) and a non-significant trend towards a decrease in those expressing VGLUT1 in the layers I/II and III of the entorhinal cortex. NAcc-DBS animals showed a significant decrease in the density of puncta expressing VGAT (p < 0.01) and VGLUT1 (p < 0.01) in the entorhinal cortex layers I/II (Fig. 5). No significant differences were found in the hippocampus (Fig. 4).

## Discussion

To our knowledge, these results are the first to date on the use of small-animal FDG-PET and neuroplasticity markers to assess the persistence of DBS modulation 1 month after stimulation withdrawal. Thus, we provide evidence that (i) the magnitude of the long-term persistence of the DBS brain metabolic modulation depends on the brain target, (ii) the modulation induced by the electrode’s presence changes over time independently of the targeted brain structure, and (iii) DBS induce neuroplasticity changes that are evident even 1 month after the stimulation ending.

FDG-PET studies revealed that LH-sham, NAcc-sham and NAcc-DBS animals showed common patterns of metabolic modulation 1 month after the end of the DBS protocol, which contrast with the metabolic pattern observed in LH-DBS rats. In fact, a reduced metabolism in striatum and somatosensory cortex, along with an increase in brainstem and hypothalamus, are evident in the former three groups of animals. Not surprisingly, all these structures are strongly interconnected through hypothalamic projections and belong to the limbic system^45^, suggesting that the lesion of a brain structure in a certain network evokes distant effects in functionally and physically connected regions. These similarities may respond to the long-term presence of the electrode and the derived lesions^6^. The absence of therapeutic effect in NAcc-DBS animals, neither during the stimulation period nor after the 1-month course^31^, as well as the fact that almost no metabolic change appeared in NAcc-DBS subjects different from those present in NAcc-sham rats, could be the clues to understand this phenomenon. In this sense, the predominance of the electrode-derived effects over the stimulation ones might respond to the need for longer stimulation periods to influence the patients’ symptoms^46^, together with a lack of strength of the stimulation impact inferred on brain networks. Together, both factors would lead to a weak cerebral modulation in the long-term, unable to produce the pursued clinical benefit. Thus, the microlesion impact on brain metabolism related to the DBS implants has been previously demonstrated^47–51^. However, to our knowledge, this is the first study in reporting continuing physiological changes derived from the electrode’s presence alone (i.e. without any influence of stimulation) as far as 2 months after implantation. Therefore, our results provide a potential explanation to the sometimes great durability of clinical outcomes related to the electrode insertional effect^52^, as this long-term metabolic modulation might be the reflect of underlying neuroplastic processes which, in some cases, may lead to important clinical benefits.

In contrast, LH-DBS rats exhibited an absence of almost any change in comparison with the previous PET session, evidencing a greater permanence of the immediate DBS effects. This finding goes in line with other authors who suggest the existence of (almost) irreversible consequences of DBS after treatment^11,13,53^. Furthermore, the sustained brain activation pattern over time could be related with the therapeutic effect, and hence the clinical benefit, observed in LH-DBS animals^17^. In this sense, Ruge et al. reported that some dystonia patients who successfully underwent DBS during several years showed a maintenance of the clinical benefits of the stimulation, even one year after turning off the device^11^. However, they also found a great neurophysiological instability under that apparent clinical calm, hypothesizing that the stimulated system would be seeking for a balance which would make the pathological symptoms evident when it was reached. Therefore, they reinforced the need to evaluate the long-term effects of a continuous DBS protocol.

Nevertheless, the ectorhinal cortex hypometabolism observed in NAcc-DBS rats suggests a certain reversibility and rebound effect of the stimulation-derived metabolic modulation, in agreement with previous reports^5,14,16^. In fact, the absence of metabolic changes in NAcc-sham animals in this region, together with the opposite effects induced by the stimulation alone in the post-1-day studies^30,31^, support this theory. Accordingly, Ewing and Grace^14^ reinforced this idea with their finding of a reduction in local field potentials towards baseline levels only 48 h after completion of high-frequency stimulation in NAcc. From the clinical side, a one-week inactivation of the stimulation in OCD patients led to a relapse of the positive symptoms and a rebound of the negative ones^16^, although this report did not specify the DBS targets stimulated or any physiological outcome under these symptomatologic features.

In addition to the regional differences in brain metabolism, DBS induced changes in neuronal plasticity patterns. The long-term effects of DBS on the expression of PSA-NCAM are indicative of ongoing changes in the circuitry. Particularly interesting are the changes observed in the hippocampus after NAcc stimulation, since both limbic regions are intensely interconnected^54,55^. Closely related to our results, Schmuckermaier et al. found that intermittent NAcc-DBS (1 h/day) during only seven consecutive days promoted neuronal activity and neurogenesis in the hippocampus, as well as antidepressant effects (i.e. improvements in the performance of the forced swimming and tail suspension tests), in a murine model of enhanced anxiety and depression^56^. In our study, the hippocampal increase in PSA-NCAM expression could be related both to changes in excitatory or inhibitory neurons, since in this limbic region, and especially in the strata of CA1 where measurements were performed, PSA-NCAM is expressed by both cell types^57^. In fact, there is a tendency towards increased levels of VGAT and VGLUT1 in the hippocampus of NAcc-DBS group.

It is also interesting the lack of metabolic changes in LH-DBS animals, and the reduction in the density of PSA-NCAM expressing puncta both in hippocampus and entorhinal cortex layer III. This could explain the low levels of VGAT obtained because PSA-NCAM is a potent regulator of inhibitory cortical networks^58^. Additionally, the most rostral region of the entorhinal cortex, as the piriform cortex layer II, harbors immature PSA-NCAM expressing neurons that progressively mature into excitatory neurons during adult life^59^, and the decrease in the expression of this molecule may boost their differentiation.

Thus, DBS could produce changes in brain regions which would trigger prolonged neuroplasticity processes in structures related to the DBS target. In this sense, although acute^60^ or early evaluations of DBS effects show more remarkable changes in brain glucose metabolism, the stimulation might drive long-lasting mechanisms of neuronal modulation which were not correlated with the metabolic state of these structures after a period without DBS. These findings match previously published effects of DBS on brain metabolism, showing that these effects seem to be transient in nature^61^ and may not be captured by FDG-PET performed 1 month after stimulation removal. Future studies should evaluate whether these changes in the expression of molecules related to neurotransmission and neural plasticity occur in other brain areas, at different times from the stimulation ending, or whether they were attenuated immediately after stimulation.

Altogether, our results support the evidence that the physiological and clinical outcomes after DBS, not only in short- but in long-terms, are strongly dependent on the brain target selected for stimulation, even under identical stimulation protocols and pathological conditions^60^. Therefore, under fixed stimulation conditions, DBS elicits the simultaneous activation of several molecular mechanisms, both at local and distant levels, which might interact but could not be gathered under a simple theoretical explanation^10^. In this sense, the length of the modulation induced by the impact of the electrical stimulation in a certain brain target would determine the persistence of the derived clinical benefits. Thus, there is a need for deeper research exploring the chronic effects of DBS, not only during the stimulation, but also after withdrawal, in order to address the complex molecular mechanisms underlying these effects.

Nonetheless, our study has certain limitations. First, our sample sizes are small, although the number of animals in each group proved to be sufficiently large to detect statistically significant changes in glucose metabolism and neuroplasticity markers. Second, our results are based on the obese Zucker rats, meaning that the currently reported results could not extrapolate to other pathological conditions. Third, our imaging data were not subjected to Bonferroni correction due to two main reasons: i) Such a strict policy as Bonferroni correction would have led to a much higher sample size, that on the other hand compromises the 3-R’s principle in animal experiments; and ii) in the case of voxel-based brain-imaging analyses, Bonferroni correction is overconservative due to assuming independence of the voxels, which is not true due to the intrinsic spatial correlation of the voxels. However, the multiple-comparison strategy followed prevents an underestimation of real effects, and hence a reduction in power^41,62^. Fourth, electrode placement for both targets may cross the lateral ventricle. On the one hand, the transventricular trajectory of the electrodes is avoided in clinical practice, as it has been related to a displacement of the electrodes. However, the stability of the electrode position throughout our study was confirmed with the three CT studies (immediately after the stereotactic surgery and before each PET study). On the other hand, transventricular trajectories have also been associated with serious neurological complications such as intraventricular hemorrhage, severe headache, postoperative confusion or seizures^63^. In this regard, none of these effects were detected in the animals included in this study. Finally, our results using an intermittent DBS protocol may differ from the possible effects of a continuous stimulation, which may lead to deeper and steadier modulatory effects than those reported here. These points highlight the need for further studies with different experimental designs in order to provide more standardized results.

## Conclusion

In conclusion, this study allowed to address the long-term persistence of DBS effects, applied in two brain structures proposed as potential targets for the treatment of obesity, 1 month after stimulation withdrawal. NAcc-DBS results supported the well-known reversibility related to DBS, only obtaining changes in those structures modulated by the DBS implants. Nevertheless, LH-DBS barely modifies the effects previously inferred to brain metabolism, and supports the relation between the preservation of the stimulation effects and the clinical benefit. Besides, the reduced expression of molecules related to neuronal plasticity in structures previously modulated by LH-DBS would support this reluctance to change. These contradictory results might possibly explain the differences in the weight gain related outcomes under both DBS protocols. Therefore, the existence of long-term effects of DBS in the brain could be understood as the result of a functional stability achieved during stimulation, which would underlie a prolonged positive outcome for the subject.

## Supplementary Information

Supplementary Information
