# Supplementary material for: Exploratory study of the long-term footprint of deep brain stimulation on brain metabolism and neuroplasticity in an animal model of obesity
Source: Sci Rep. 2021 Mar 10;11:5580. doi: 10.1038/s41598-021-82987-7 (PMC7946931; doi:10.1038/s41598-021-82987-7)
Supplement: Supplementary file 1 — Supplementary Information [file 41598_2021_82987_MOESM1_ESM.pdf]

Supplementary material

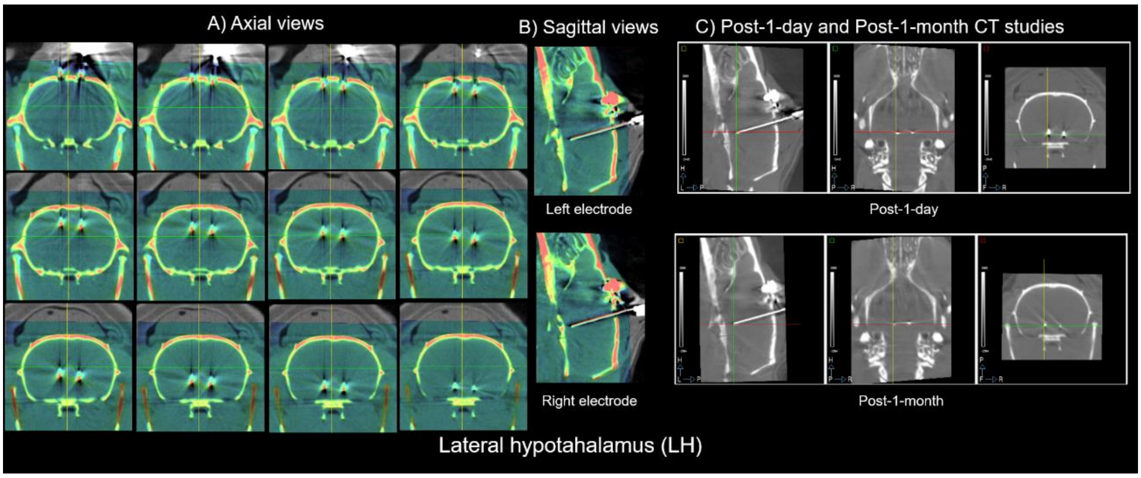

Supplementary Fig 1. Verification of absence of electrode displacement in the long-term in one LH operated animal. A) and B) show the post-1-month CT overlaid on the post-1-day CT of the same animal with electrodes targetting the LH. C) Represents the sagital, coronal and axial views of the post-1-day and post-1-month CT studies for the same rat presented in A) and B).

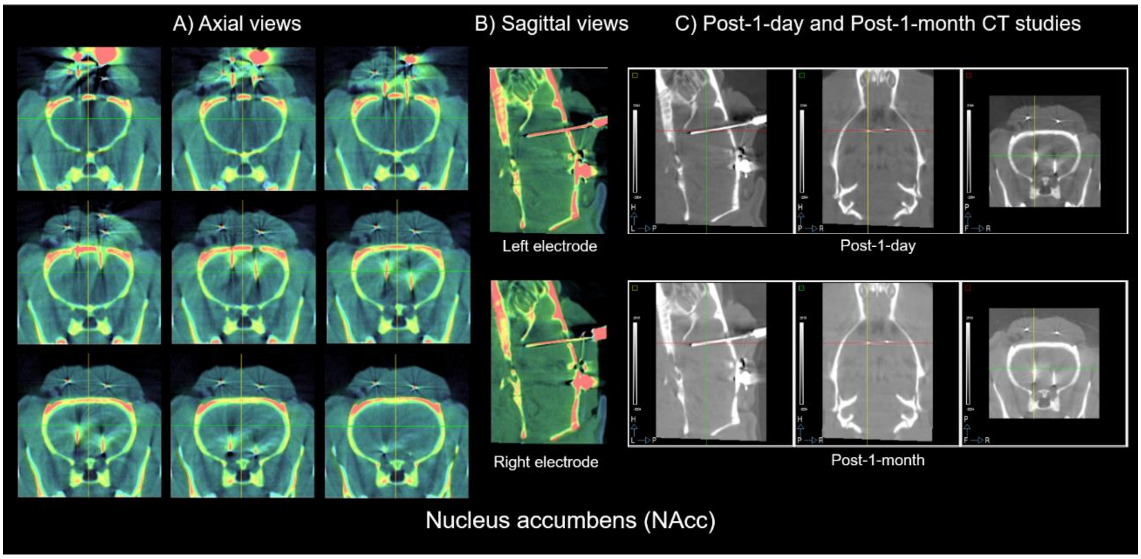

Supplementary Fig 2. Verification of absence of electrode displacement in the long-term in one NAcc-operated animal. A) and B) show the post-1-month CT overlaid on the post-1-day CT of the same animal with electrodes targetting the NAcc. C) Represents the sagital, coronal and axial views of the post-1-day and post-1-month CT studies for the same rat presented in A) and B).

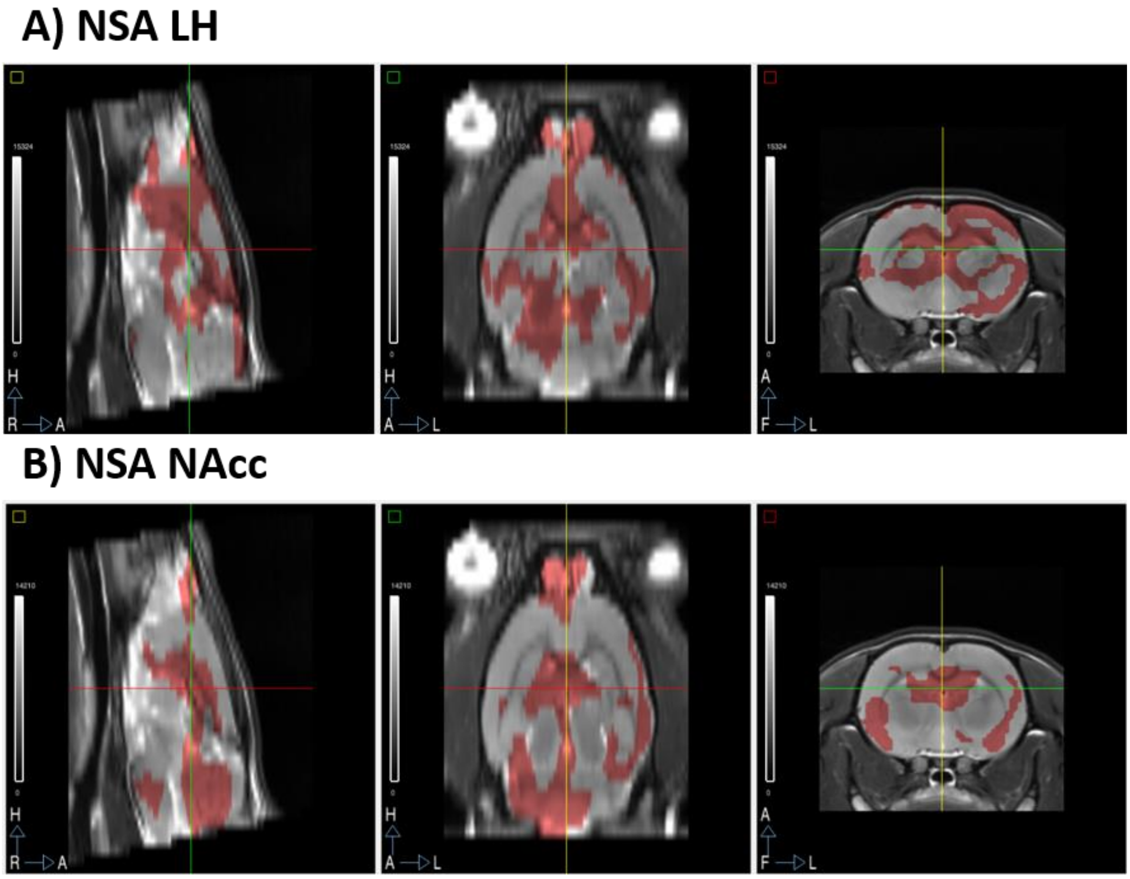

Supplementary Fig 3. Sagittal, coronal and axial views of the NSA masks for SPM analyses. A) Final NSA mask (red colored voxels) for the LH analyses overlaid on the reference MR image. B) Final NSA mask (red colored voxels) for the NAcc analyses overlaid on the reference MR image.
